# Plasma Metabolic Profiles of Chronic and Recurrent Uveitis Treated by Artesunate in Lewis Rats

**DOI:** 10.3390/biomedicines13040821

**Published:** 2025-03-28

**Authors:** Xinyi Gong, Jingchuan Fan, Hui Huang, Fei Xu, Kaijiao Hu, Jianping Liu, Yi Tan, Feilan Chen

**Affiliations:** 1 Laboratory Animal Center, Chongqing Medical University, Chongqing 400016, China; gongxinyi0308@163.com (X.G.); huanghui290077@163.com (H.H.); xfei163com@163.com (F.X.); 17835417808@163.com (K.H.); 2 Chongqing Engineering Research Center for Rodent Laboratory Animals, Chongqing 400016, China; 3 Institute of Life Sciences, Chongqing Medical University, Chongqing 400016, China; fanjc@cqmu.edu.cn; 4 Department of Pathology, Chongqing Medical University, Chongqing 400016, China; L15223336386@163.com

**Keywords:** recurrent autoimmune uveitis, artesunate, metabolomics, metabolic pathways, prognosis

## Abstract

**Background/Objectives**: Identifying effective and safe treatment options for non-infectious uveitis remains challenging due to chronic and relapsing ocular inflammation. Previous studies have shown that artesunate (ART) plays an immunosuppressive role in several classic autoimmune diseases, including uveitis. However, its impact on the plasma metabolic profile of recurrent autoimmune uveitis remains unclear. This study aims to explore the effect of ART on the plasma metabolic features of recurrent experimental autoimmune uveitis (EAU) in a Lewis rat. **Methods**: Rats were clinically and pathologically evaluated for the development of recurrent EAU induced by inter-photoreceptor retinoid-binding protein (IRBP) R16 peptide-specific T-cells (tEAU). The disruptive effects of ART on tEAU were investigated to evaluate the potential role of rat recurrent EAU. Differentially expressed metabolites were identified in the plasma of rats by untargeted metabolomics analysis after ART treatment. The differential metabolites were applied to subsequent pathway analysis and biomarker analysis by MetaboAnalyst. **Results**: ART can significantly alleviate the severity of clinical signs and pathological injuries of eyeballs with tEAU. Both non-supervised principal component analysis and orthogonal partial least-squares discriminant analysis showed 84 differential metabolites enriched in 16 metabolic pathways in the tEAU group compared with heathy controls and 51 differential metabolites enriched in 17 metabolic pathways, including arginine and proline metabolism, alanine metabolism, and aminoacyl-tRNA biosynthesis, in the ART-treated group compared with the tEAU group. Particularly, upregulated L-alanine levels in both alanine metabolism and aminoacyl-tRNA biosynthesis were associated with T-cell activation, while elevated spermidine and N-acetyl putrescine levels in arginine and proline metabolism related to T-cell differentiation proved to be valuable biomarkers for ART treatment. **Conclusions**: Our study demonstrates that ART treatment can alleviate recurrent uveitis by altering the plasma metabolic characteristics associated with T-cell activation and differentiation, which might provide novel insights for potential therapeutic treatments.

## 1. Introduction

Autoimmune uveitis represents a group of ocular inflammatory diseases caused by autoimmune responses targeting antigens in the retina and/or choroid and is a key cause of severe vision impairment [1]. In patients with autoimmune uveitis, the persisting and relapsing inflammation in ocular tissue is difficult to cure and therefore leads to neural retinal damage and ultimately vision loss [2].

The most common treatment strategies for autoimmune uveitis consist of conventional immunosuppressive drugs, such as corticosteroids and biomedical reagents, and anti-inflammation therapies. However, most patients with uveitis are refractory to these conventional drugs and suffer serious side effects after long-term use. Artesunate (ART), a water-soluble analog of artemisinin with a long half-life and low toxicity, has been proven to be a safe and effective treatment for malaria [3]. In addition to its classic antimalarial abilities, clinical and experimental studies have shown that ART has regulatory effects in several autoimmune diseases, including lupus erythematosus, multiple sclerosis, rheumatoid arthritis, and inflammatory bowel disease [4,5,6,7]. A pilot clinical study showed that ART can effectively ameliorate ocular neovascularization in humans [8]. Meanwhile, it has been demonstrated that artemisinin and its derivatives can prevent or improve acute inflammation in endotoxin-induced uveitis in Long–Evans rats [9]. However, the effect of ART on the plasma metabolic features of recurrent autoimmune uveitis (EAU) remains to be elucidated.

In the present study, ART treatment effects on relapsing uveitis induced by interphotoreceptor retinoid-binding protein (IRBP) R16 peptide-specific T-cells (tEAU) to Lewis rats were investigated, and plasma differential metabolic profiles were subsequently explored after ART treatment. The results showed that ART could alleviate the severity of clinical signs and histopathological lesions of the rat eyeball with tEAU. Particularly, upregulated L-alanine levels in alanine metabolism associated with T-cell activation and aminoacyl-tRNA biosynthesis, as well as increased spermidine and N-acetyl putrescine levels in arginine and proline metabolism associated with T-cell differentiation, were realized in significant fashion by ART treatment.

## 2. Materials and Methods

### 2.1. Reagents and Animals

ART was purchased from Shanghai Aladdin Biochemical Technology Co., Ltd. (Shanghai, China). Bovine R16 peptide derived from inter-photoreceptor retinoid-binding protein (IRBP) (amino acids 1177–1191; ADGSSWEGVGVVPDV) was synthesized by China Peptides Co., Ltd. (Shanghai, China). Specific-pathogen-free (SPF) female Lewis rats aged 6–8 weeks were purchased from Vital River Laboratory Animal Technology Co., Ltd. (Beijing, China) and maintained at the animal facilities of Chongqing Medical University, and they were given free access to chow and water. All procedures were carried out in compliance with the guidelines of the National Institutes of Health, the ARVO Statement, and the ARRIVE Guidelines for the Use of Animals in Ophthalmic and Vision Research to ensure the appropriate care and use of animals [10]. The protocol for the animal studies was approved by the Ethics Committee of Chongqing Medical University (permit no. 2021071).

### 2.2. Animal Model of EAU and Drug Treatment

#### 2.2.1. Induction of Antigen-Induced Uveitis (aEAU)

For the development of retinal aEAU in Lewis rats, bovine peptide R16 was emulsified and immunized into female Lewis rats according to previous protocols [11] with slight modification. Briefly, the rats were immunized with 200 µL of an emulsion containing 100 µg of R16 and 500 µg of Mycobacterium tuberculosis H37Ra (H37RA, ATCC 25177; American Type Culture Collection, Manassas, VA, USA) in Complete Freund’s Adjuvant (CFA, St. Louis, MO, USA), distributed over four spots on the tail base and flank.

#### 2.2.2. Induction of Adoptive tEAU and Drug Treatment

Induction of uveitis by adoptive transfer of R16-specific T-cells into naive Lewis rats was performed as previously described [11]. Briefly, the uveitogenic T-cells and antigen-presenting cells (APCs) were isolated from draining lymph nodes or spleens of aEAU rats immunized with R16 on day 11 post-immunization and enriched using nylon wool based on previous studies [11]. T-cells were then stimulated with 20 µg/mL of R16 in the presence of syngeneic APC in RPMI 1640 medium (Gibco, Grand Island, NY, USA) supplemented with 10% fetal calf serum (Gibco, Grand Island, NY, USA), 0.1% mercaptoethanol, and 1% penicillin/streptomycin at 37 °C in 5% CO_2_ for 56–72 h. Subsequently, the activated T-cells were isolated by gradient centrifugation in mouse 1× lymphocyte separation medium (Dakewe Biotech Co., Ltd., Shenzhen, China) according to the operating manual. For adoptive transfer, recipient rats (8–10 weeks old) were intraperitoneally injected with 3 × 10^7^ R16-specific T-cells in 0.2 mL of phosphate-buffered saline.

To investigate the effect of ART on tEAU, rats were randomly divided into an ART-treated group, sodium bicarbonate (SOD)-treated group, tEAU model group, and healthy control group. Then, 3 or 4 rats were treated with 30 mg/kg of ART dissolved in 5% SOD in 1000 µL of 5% phosphate-buffered saline intraperitoneally every day from days −3 to 19 or 20 days post-immunization. Another 3–5 rats were treated with 30 mg/kg of 5% SOD in 1000 µL of phosphate-buffered saline intraperitoneally every day from days −3 to 19 or 20 days post-immunization. Separately, 3–4 rats in the tEAU model were only adoptively treated with R16-specific T-cells without drug treatment, and 3–4 healthy control rats were clinically investigated without T-cell transfer or drug treatment.

### 2.3. Clinical Evaluation

The immunized rats were examined once every 2 days by slit light microscopy (Sunshine Medical Equipment Co., Ltd., Chongqing, China) at a magnification of 40× to record the clinical signs from day 3 post-immunization. The intensity of uveitis in each eye was scored from days 3 to 18 or 19 after T-cell transfer in a blind manner on an arbitrary scale of 0–4 points as previously described [11]. The EAU scoring system used was as follows: 0 points, normal; 0.5 points, dilated blood vessels in the iris; 1 point, abnormal pupil contraction; 2 points, hazy anterior chamber; 3 points, moderately opaque anterior chamber but pupil still visible; and 4 points, opaque anterior chamber and obscured pupil. During clinical sign observation, rats showing an inability to obtain food or water were euthanized as humane endpoints. Meanwhile, an antibiotic ointment was applied if ulcers occurred on the skin of the immunization sites.

### 2.4. Histological Examinations

The rats were euthanized while significantly reduced clinical signs were observed after ART treatment on day 19 or 20 after immunization. Subsequently, their eyeballs were collected and fixed in eyeball liquid (Powerful Biology, Wuhan, China) for histopathological evaluation. Paraffin-embedded eye tissues were sliced into 4 μm thick sections for hematoxylin and eosin staining. The HE-stained sections were observed under a 100× optical microscope (Leica DM2000, Leica Microsystems Ltd., Wetzlar, Germany), and the tissue images were taken with Leica Application Suite X (LAS X, Leica Microsystems Ltd., Wetzlar, Germany). Histopathological grades were evaluated in a masked manner in accordance with the reported criteria in the literature [11].

### 2.5. Plasma Untargeted Metabolomics Analysis

#### 2.5.1. Collection of Plasma Samples

When the alleviated clinical appearance of rat eyes after ART treatment was observed, blood samples were collected in the tube with EDTA anticoagulant. Plasma was obtained from blood by centrifuge at 2000 rpm for 10 min and stored in the supernatant at −80 °C until analysis. A total of 100 μL of the plasma sample was added to 400 μL of a mixture of cold methanol and acetonitrile (1:1, *v*/*v*), then vortexed and placed at −20 °C for 4 h, which was followed by centrifugation at 12,000 rpm for 12 min at 4 °C. Subsequently, the supernatant was processed in a vacuum freezer dryer, diluted with ultrapure water to achieve 50% acetonitrile content before being centrifuged at 12,000 rpm for 5 min at 4 °C. Finally, the supernatant was collected as the testing sample. Quality control (QC) samples consisted of a mixture of 10 μL of all 32 samples in the four groups. The pooled QC samples were injected at the beginning, at the end, and at every eight samples throughout the analytical run to verify the reliability of the whole procedure. A reproducibility of the peak chromatogram area between each pair of QC samples of <25% and a time sustained at <0.1 min were regarded as indicating a qualified sample.

#### 2.5.2. Ultra-High Performance Liquid Chromatography (UHPLC)–Quadrupole Time-of-Fight (TOF) Mass Spectrometry (UHPLC-MS/MS) Analysis

After the pretreatment of plasma and the qualified control evaluation, a hybrid Quadrupole-TOF LC coupled with MS (Triple TOF 6600 mass spectrometer system; AB Sciex, Framingham, MA, USA) was performed in both positive and negative ion modes. For chromatographic separation, ACQUITY UPLC BEH Amide 1.7-μm columns (100 mm × 2.1 mm; Waters Corp., Milford, MA, USA) and ACQUITY UPLC HSS T3 1.8-μm columns (100 mm × 2.1 mm; Waters Corp., Milford, MA, USA) were utilized in both positive and negative modes for subsequent sample analyses. The mobile phase of BEH Amide 1.7 μm columns was set as follows: mobile phase A = 5 mmol/L of ammonium formate containing 0.02% formic acid and mobile phase B = 95% acetonitrile in water. The elution procedure was set as follows: after stabilizing it for 2 min, the gradient of 95% B was linearly decreased to 70% in 9 min; then, it was reduced to 30% in 2 min. Finally, after stabilizing 30% B for 2 min, it was increased to 100% in 10 s. A 350 s re-equilibration period was used in the procedure. The sampling rate was set as 5.0 μL/s at 10 °C. The column oven temperature was set at 35 °C, and 2 μL plasma specimens were injected into the column. The mobile phase of the T3 column was set as follows: mobile phase A = 5 mmol/L of ammonium formate containing 0.05% formic acid and mobile phase B = 100% acetonitrile. The elution procedure was set as follows: the gradient of 2% B was increased to 20% in 1.5 min, then increased to 65% in 2.5 min and continually increased to 95% in 7 min. After stabilizing 95% B for 3 min, it was increased to 100% in 10 s. After stabilizing 100% B for 170 s, it was reduced to 2% in 10 s and maintained as such for 170 s. The sampling rate was set at 5.0 μL/s at 10 °C. The column oven temperature was set at 40 °C, and a 3 μL plasma specimen was injected into the column.

The Triple TOF 6600 mass spectrometer system was used in both positive and negative ion modes according to the following running circumstances and parameters. Briefly, the settings of the mass spectrometer were set as follows: ion gas 1 = 60 psi; ion gas 2 = 60 psi; curtain gas = 35 psi; temperature = 600 °C; and floating ion spray voltage = 5500 eV. The MS/MS spectra were attained by using information-dependent acquisition coupled with the selected high-sensitivity mode. The parameters were set as follows: collision energy = 50 eV (+) and −20 eV (−), with declustering potential = 60 V (+) and −60 V (−), excluding isotopes within 4 Da and with candidate ions to monitor per cycle = 6. MS1 had a mass range of 80–100, while MS2 had a mass range of 50–1100. The accumulation time of both MS1 and MS2 was set at 18 min. Each sample was run in triplicate.

### 2.6. Metabolomics Data Processing

The original data were converted to mzXML format using the ProteoWizard MSConvert GUI (64-bit). The converted format data were imported into the MarkerView 1.3.1 software (AB Sciex Pte., Ltd., Framingham, MA, USA) for peak extraction, peak grouping, and peak alignment and saved in EXCEL format for subsequent analysis. The metabolomic data were analyzed by group comparisons, which included the healthy control group vs. the tEAU group, the ART-treated group vs. the tEAU group, the ART-treated group vs. the SOD-treated group, and the SOD-treated group vs. the tEAU group, by Metaboanalyst 5.0 (Wishart Research Group, University of Alberta, Edmonton, Canada https://www.metaboanalyst.ca/ (accessed on 21 October 2022, 13 December 2022 and 24 February 2025)). Metabolites with *p* < 0.05 and VIP > 1 were considered significantly altered. Non-supervised principal component analysis (PCA) and orthogonal partial least-squares discriminant analysis (OPLS-DA) were then performed to characterize the differential profiles between those matched groups. Metabolite identification was conducted based on the MetDNA2 database. Finally, MetaboAnalyst 5.0 was used to perform pathway enrichment analysis and biomarker analysis.

### 2.7. Statistical Analysis

The animal experiments were conducted three times to confirm the experimental data. Statistical analysis was performed using GraphPad Prism 8.0.1 for Windows (GraphPad Software, Inc., San Diego, CA, USA). Non-parametric statistical tests (Kruskal–Wallis test) or unpaired Student’s *t* test were carried out where appropriate. Statistical significance was set at *p* < 0.05.

## 3. Results

### 3.1. Alleviated Clinical Appearance of tEAU After ART Treatment

**Figure 1:**
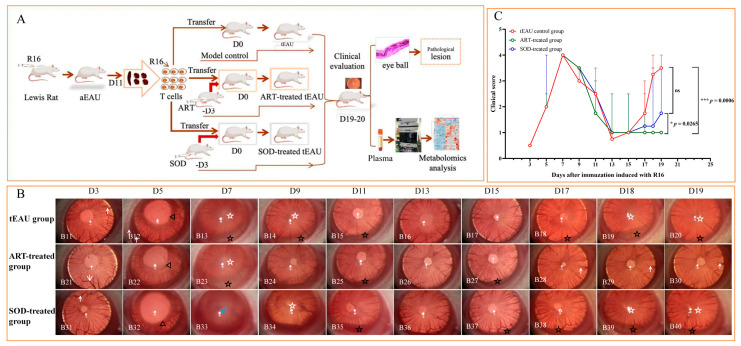
Study design and clinical evaluations of Lewis rat tEAU in different groups. (**A**) Schematic illustration of the experimental design. First, bovine peptide R16 emulsified in Complete Freund’s Adjuvant (CFA) was subcutaneously injected into Lewis rats to induce antigen-specific uveitis (aEAU). Subsequently, R16-specific T-cells from the spleen and lymph nodes of rats with aEAU on day 11 post-immunization were adoptively transferred into a naïve rat to induce recurrent uveitis (tEAU). To study the therapeutic roles of artesunate (ART), rats were treated with 30 mg/kg of ART intraperitoneally every day from day −3 through days 19 or 20 post-T-cell-transfer. The sodium bicarbonate (SOD)-treated group and tEAU model group were designated as the solution comparison group and model comparison group, respectively. Clinical signs were observed and graded beginning on day 3 after T-cell transfer once every 2 days until a significant alleviation of clinical signs was observed. Finally, rats were sacrificed for histopathology of the eyeball and plasma metabolomics analysis. (**B**) Rats in the ART treatment group presented milder clinical signs during the second inflammation relapse than the other groups. The disease course between these groups was similar during the first inflammation phase (**B11**–**B16**,**B21**–**B26**,**B31**–**B36**), including onset on day 3, peaking on day 7, and a gradual remission 13 days post-T-cell transfer. However, the relapses in ART-treated rats occurred earlier and were milder on day 15, followed by a prompt recovery until day 19 after T-cell transfer (**B27**–**B30**) compared with the tEAU group (**B17**–**B20**) and SOD-treated group (**B37**–**B40**). (**C**) Average clinical scores per eye with tEAU are shown from groups with transferred T-cells. There was a reduced clinical score in the ART-treated group during the recurrent inflammation on day 19 after T-cell transfer compared to the tEAU group and SOD-treated group. Data are shown as the median ± error of the median (n = 8; “n” is the number of eyes) and are representative of three independent experiments (Kruskal–Wallis test), * *p* < 0.05, *** *p* < 0.001.

Firstly, we evaluated whether ART would inhibit the clinical appearance of tEAU in Lewis rats (Figure 1A). As shown in Figure 1B, onset of clinical disease was observed 3 to 13 days after T-cell transfer in the ART-treated group (Figure 1(B21–B26)), the tEAU model group (Figure 1(B11–B16)), and the SOD-treated group (Figure 1(B31–B36)). All three groups presented with similar features, such as dilated blood vessels in the iris (short arrow), abnormal pupil contraction (triangle), a hazy anterior chamber (black star), a moderately opaque anterior chamber (white star) with the pupil still visible, and an opaque anterior chamber with an obscured pupil (blue star). The disease course between these groups was similar during the first inflammation phase, including onset on day 3, peaking on day 7, and gradual remission 13 days post-T-cell transfer. However, the relapses in ART-treated rats occurred earlier and were milder on day 15, followed by prompt recovery until day 19 after T-cell transfer (Figure 1(B27–B30)) compared with the tEAU group (Figure 1(B17–B20)) and SOD-treated group (Figure 1(B37–B40)). Meanwhile, the clinical scores of tEAU in the ART-treated group were significantly decreased at the recurrent point on days 18–19 post-immunization compared with the tEAU model group (*p* = 0.0006, Kruskal–Wallis test, Uncorrected Dunn’s test). Furthermore, clinical scores in the ART-treated group demonstrated a significant reduction compared with the SOD-treated group at the same timepoint (*p* = 0.0265, Kruskal–Wallis test, Uncorrected Dunn’s test). Notably, the SOD-treated group showed no statistically significant differences relative to the tEAU model group (*p* = 0.1579, Kruskal–Wallis test, Uncorrected Dunn’s test) (Figure 1C); this finding suggest that the SOD treatment exerted minimal impact on the clinical manifestations of tEAU.

### 3.2. Reduced Histopathological Lesions of tEAU After ART Treatment

**Figure 2:**
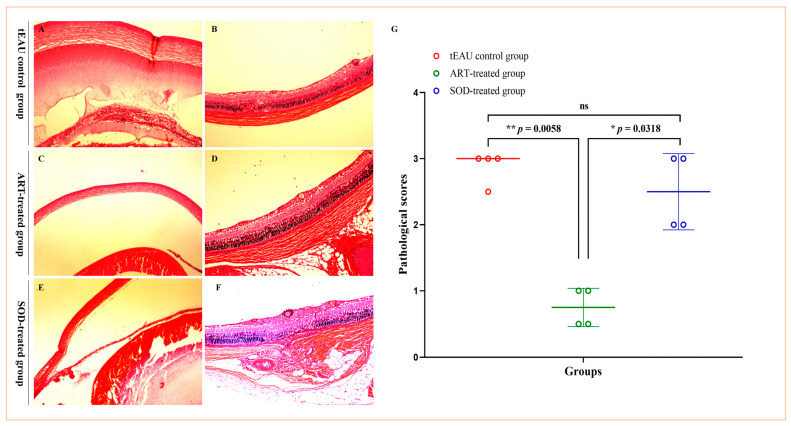
Histopathological evaluations of the control and ART-treated rats on day 18 following T-cell transfer. Histological images of the artesunate (ART)-treated group (**C**,**D**) showed decreased inflammation in the anterior chamber and retinal layer compared to the recurrent uveitis induced by IRBP R16 peptide-specific T-cells (tEAU) model group (**A**,**B**) and sodium bicarbonate (SOD)-treated group (**E**,**F**), as observed via hematoxylin and eosin (H&E) staining at 100 magnification. (**G**) The ART-treated group had a reduced histopathological score compared to the tEAU model group and SOD-treated group. Histological scores were evaluated on sections stained with H&E from the three groups of rats. Data are shown as the median ± error of the median (n = 4) and are representative of three independent experiments. * *p* < 0.05, ** *p* < 0.01 based on the Kruskal–Wallis test.

While observing the alleviation of clinical signs on days 18–19 post-immunization after ART treatment, eyeballs were collected for further evaluation of the histopathological effects of ART treatment on tEAU rats. Mild inflammatory infiltration in the anterior segment and photoreceptor cell damage (Figure 2C,D) were present in the ART-treated rats, while moderate or severe inflammatory infiltration in the anterior chamber and retinal damage were observed in the tEAU and SOD-treated groups (Figure 2A,B,E,F). Moreover, significantly reduced pathological scores in the ART-treated group were recorded by comparison with the tEAU control group and SOD-treated group (Figure 2G; *p* = 0.0058 vs. tEAU control group; *p* = 0.0318 vs. SOD-treated group, Kruskal–Wallis test, Uncorrected Dunn’s test). No significant differences were observed between the SOD-treated group and the tEAU control group (Figure 2G; *p* = 0.5395, Kruskal–Wallis test, Uncorrected Dunn’s test); this result indicates that SOD treatment had a minimal effect on the pathological phenotype of tEAU.

### 3.3. Altered Plasma Metabolomic Profiles After ART Treatment

We further performed untargeted plasma metabolomics profile analysis to explore the mechanism of ART treatment on recurrent autoimmune uveitis. The chromatograms of HILIC-MS and RPLC-MS of the QC samples in positive and negative ion modes are shown in Figure S1. Throughout the test, the stability of the machine was judged by observing the chromatogram of the QC sample. There was no QC deviation out of bounds throughout the whole process, suggesting that the stability and repeatability of the instrument were good and the data of this batch of samples were reliable. Subsequently, all samples were analyzed in the positive and negative ion modes of HILIC-MS (Figure S1A,B) and RPLC-MS (Figure S1C,D), and a base peak chromatogram was generated.

**Figure 3:**
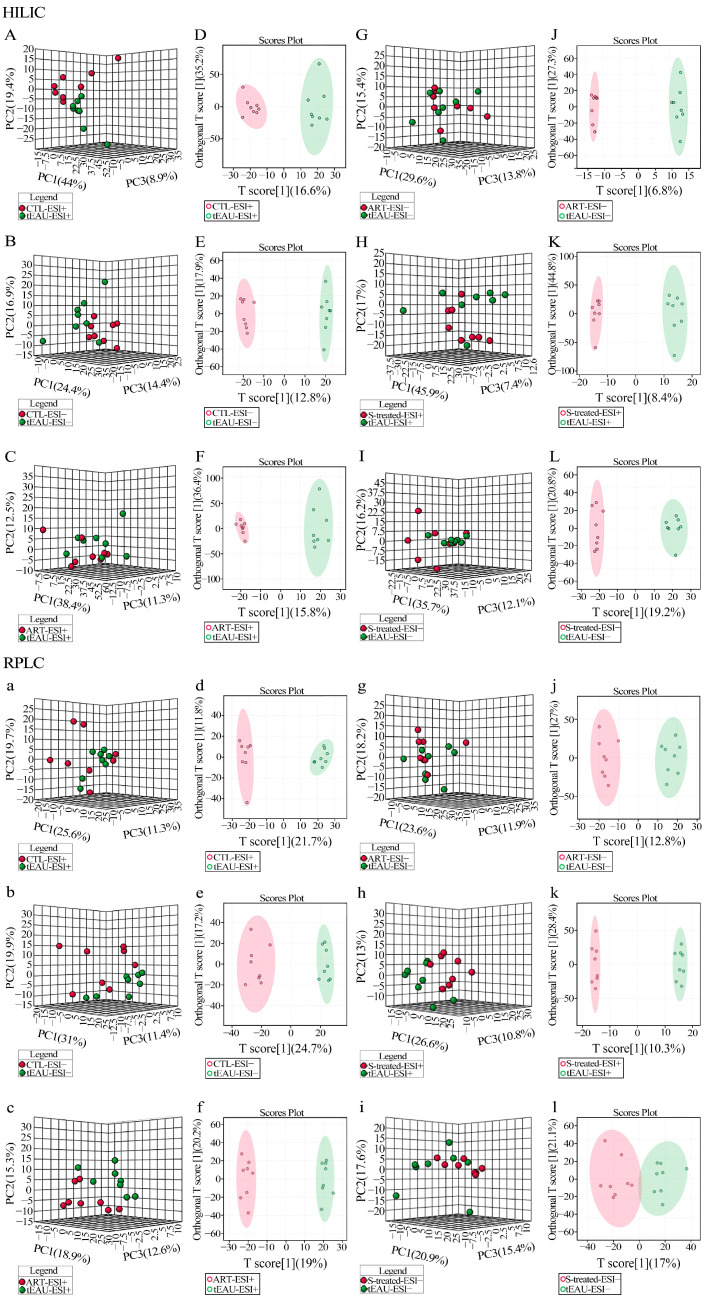
Untargeted metabolomics MetaboAnalyst PCA and OPLS-DA plot. Plasma metabolomics analysis of samples from the recurrent uveitis induced by IRBP R16 peptide-specific T-cells (tEAU) model (n = 8), artesunate (ART)-treated (n = 8), and sodium bicarbonate (SOD/ S)-treated (n = 8) groups in the HILIC column (**top**) and the RPLC column (**bottom**). Principal component analysis (PCA) showed discrimination based on the plasma metabolomic profile. CTL-ESI+ vs. tEAU-ESI+ (**A**,**a**); CTL-ESI− vs. tEAU-ESI− (**B**,**b**); ART-ESI+ vs. tEAU-ESI+ (**C,c**); ART-ESI− vs. tEAU-ESI− (**G**,**g**); SOD-treated-ESI+ vs. tEAU-ESI+ (**H**,**h**); SOD-treated-ESI− vs. tEAU-ESI− (**I**,**i**). Orthogonal partial least-squares discriminant analysis (OPLS-DA) showed significant discrimination based on the plasma metabolomic profile. CTL-ESI+ vs. tEAU-ESI+ (**D**,**d**); CTL-ESI− vs. tEAU-ESI− (**E**,**e**); ART-ESI+ vs. tEAU-ESI+ (**F**,**f**); ART-ESI− vs. tEAU-ESI− (**J**,**j**); SOD-treated-ESI+ vs. tEAU-ESI+ (**K**,**k**); SOD-treated-ESI− vs. tEAU-ESI− (**L**,**l**). Healthy control group (CTL).

**Figure 4:**
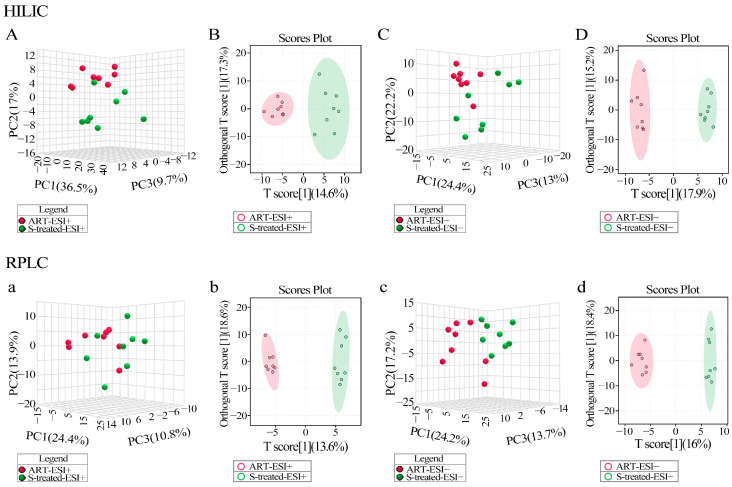
PCA and OPLS-DA of untargeted metabolomics profiling between ART- and SOD-treated groups (MetaboAnalyst). Plasma metabolomics analysis of samples from the artesunate (ART)-treated (n = 8), and sodium bicarbonate (SOD/S)-treated (n = 8) groups in the HILIC column (**top**) and the RPLC column (**bottom**). Principal component analysis (PCA) showed discrimination based on the plasma metabolomic profile. ART-ESI+ vs. S-treated-ESI+ (**A**,**a**); ART-ESI− vs. S-treated-ESI− (**C**,**c**). Orthogonal partial least-squares discriminant analysis (OPLS-DA) showed significant discrimination based on the plasma metabolomic profile. ART-ESI+ vs. S-treated-ESI+ (**B**,**b**); ART-ESI− vs. S-treated-ESI− (**D**,**d**).

A comparison of the healthy control (CTL) group vs. tEAU model group, ART-treated group vs. tEAU model group and SOD-treated group vs. tEAU model group revealed a remarkable separation via OPLS-DA and PCA in the HILIC column (Top, Figure 3A–L) and the RPLC column (Bottom, Figure 3a–l). In addition, we also performed PCA and OPLS-DA analyses on the ART-treated group vs. the SOD-treated group. Similarly, distinct groups could be clearly distinguished in both the HILIC column (Top, Figure 4A–D) and the RPLC column (Bottom, Figure 4a–d).

#### Altered Metabolomic Profiles of Plasma in the tEAU Rat Model After ART Treatment

**Figure 5:**
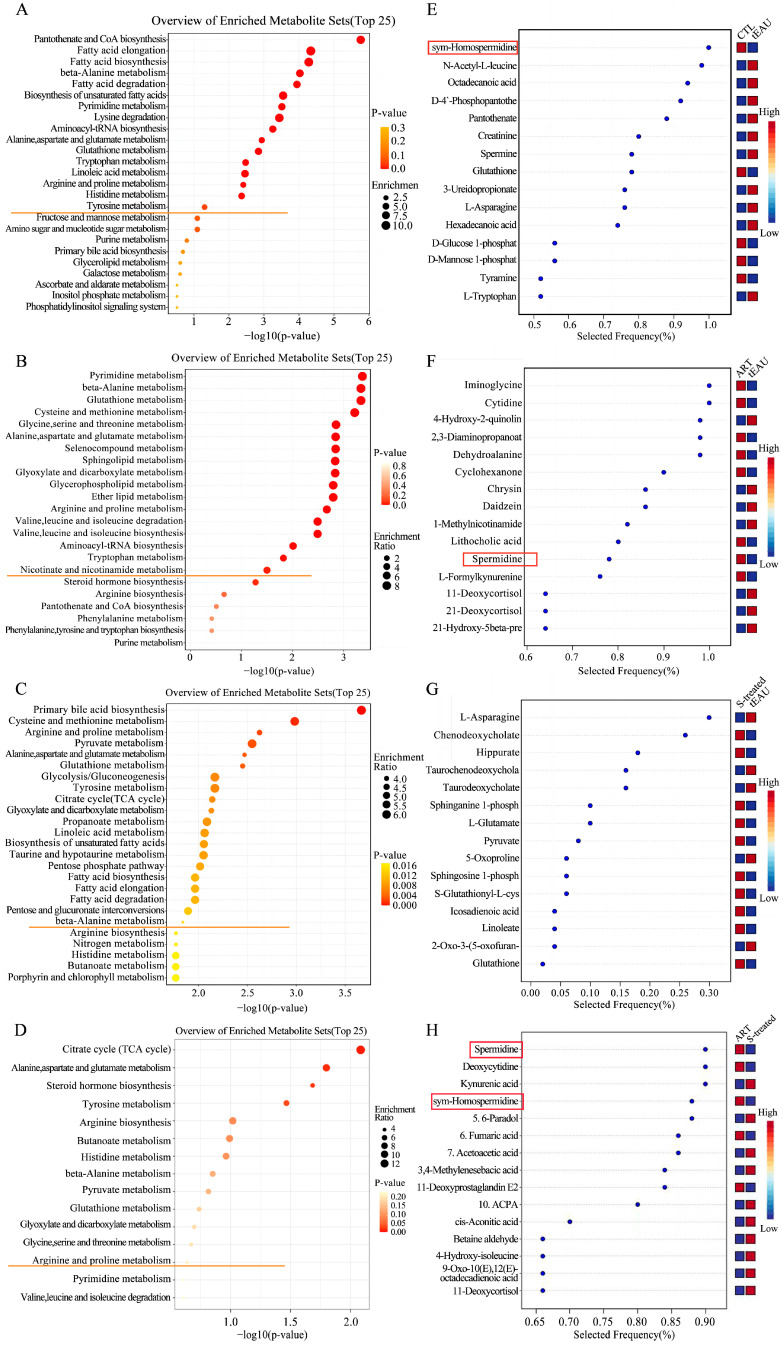
KEGG analysis of the differentially expressed metabolites and the metabolic biomarkers between the matched groups. The significantly enriched pathways are above the straight orange line (**A**–**D**), while significantly altered metabolites are represented by red rectangles (**E**–**H**). (**A**,**E**): CTL group (n = 8) vs. tEAU group (n = 8). (**B**,**F**): ART-treated group (n = 8) vs. tEAU group (n = 8). (**C**,**G**): SOD-treated group (n = 8) vs. tEAU group (n = 8). (**D**,**H**): ART-treated group (n = 8) vs. SOD-treated group (n = 8). Healthy control group (CTL); recurrent uveitis induced by IRBP R16 peptide-specific T-cells (tEAU); artesunate (ART); sodium bicarbonate (SOD).

**Figure 6:**
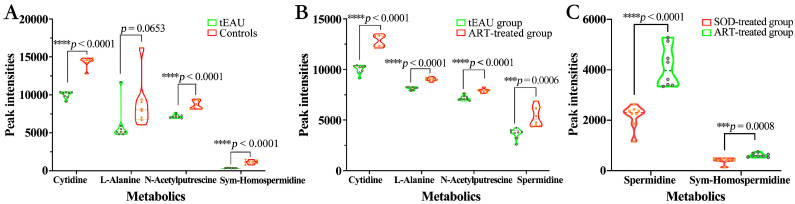
Expression level of the discriminant metabolites between the matched groups. ART treatment significantly enhances the level of discriminant cytidine, L-alanine, N-acetyl putrescine, and spermidine in the tEAU rats. (**A**): CTL group (n = 8) vs. tEAU group (n = 8). (**B**): ART-treated group (n = 8) vs. tEAU group (n = 8). (**C**): ART-treated group (n = 8) vs. SOD-treated group (n = 8). *** *p* < 0.001, **** *p* < 0.0001 based on the unpaired Student’s *t* test. Artesunate (ART); recurrent uveitis induced by IRBP R16 peptide-specific T-cells (tEAU); healthy control group (CTL); sodium bicarbonate (SOD).

A total of 84 significantly altered metabolites were identified in the plasma of the tEAU group, including 17 upregulated metabolites and 67 downregulated metabolites, compared to the healthy control group. Furthermore, those discriminative metabolites were significantly enriched in 16 pathways, including pyrimidine metabolism; glutathione metabolism; alanine, aspartate, and glutamate metabolism; and arginine and proline metabolism (Figure 5A). Specifically, downregulated L-alanine levels related to alanine, aspartate, and glutamate metabolism and downregulated N-acetyl putrescine and sym-homospermidine levels related to arginine and proline metabolism in the tEAU group were identified compared with levels in the healthy control group (Table S1 and Figure 6A). Furthermore, a total of 51 significantly altered metabolites were identified in the plasma in a comparison between the tEAU and ART-treated groups, and 20 upregulated metabolites and 31 downregulated metabolites in the ART-treated group were identified by comparison with the tEAU group. The 17 markedly enriched pathways were analyzed, including those of alanine, aspartate, and glutamate metabolism and arginine and proline metabolism. Particularly, upregulated L-alanine levels of alanine, aspartate, and glutamate metabolism and upregulated spermidine and N-acetyl putrescine levels of arginine and proline metabolism in the ART-treated group were identified when compared with the tEAU group (Figure 5B and Figure 6B). These results suggest that ART plays a suppressive role in relapsing uveitis by upregulating L-alanine involved in alanine, aspartate, and glutamate metabolism and spermidine and N-acetyl putrescine related to arginine and proline metabolism, respectively.

In addition, a total of 49 significantly altered metabolites, including 32 increased metabolites and 17 decreased metabolites, enriched in 21 essential pathways were identified in the plasma of the SOD-treated group compared with the tEAU group (Figure 5C).

Of special interest, there are six shared enriched pathways among the tEAU, ART-treated, and SOD-treated groups compared with the matched control group, including pyrimidine metabolism; beta-alanine metabolism; glutathione metabolism; alanine, aspartate, and glutamate metabolism; arginine and proline metabolism; and aminoacyl-tRNA biosynthesis. Moreover, opposing levels of L-alanine, spermidine, and N-acetyl putrescine associated with alanine, aspartate, and glutamate metabolism; aminoacyl-tRNA biosynthesis; and arginine and proline metabolism were found to exist in the comparison of the ART and tEAU groups with the matched controls. However, the distinct differential metabolites were impacted in the comparison of ART and SOD to the matched controls, suggesting that both groups have different routes of treatment.

Comparative metabolomics between ART- and SOD-treated groups identified 23 significantly altered plasma metabolites (6 upregulated, 17 downregulated). These differential metabolites were enriched in 15 metabolic pathways, including alanine, aspartate, and glutamate metabolism, β-alanine metabolism, and arginine and proline metabolism (Figure 5D). Notably, spermidine levels in the arginine and proline metabolism showed significant elevation in the ART-treated group compared to the SOD-treated group (Figure 6C). These results demonstrate that ART exerts inhibitory effects on recurrent uveitis by specifically upregulating spermidine biosynthesis within the arginine–proline metabolic axis, with minimal interference from SOD treatment.

Finally, we conducted biomarker analysis (multivariate exploratory receiver operating characteristic curve analysis overview) to explore the potential biomarkers in relapsing uveitis and ART treatment. As shown in Figure 5E–H, spermidine was upregulated in the ART group but downregulated in the tEAU group and SOD group, suggesting that spermidine could be a potential predictive therapeutic biomarker in ART treatment.

## 4. Discussion

In the current study, we evaluated the therapeutic role of the classical anti-malaria drug ART on recurrent uveitis and differential metabolic profile mechanisms in a rat model. Our results revealed that ART could reduce the severity of clinical signs and pathological lesions, but the inflammation recurred on days 18–19 post-immunization. ART treatment in tEAU rats can alter the characteristic metabolic profiles, especially the three enriched pathways of alanine, aspartate, and glutamate metabolism; arginine and proline metabolism; and aminoacyl-tRNA biosynthesis.

Clinical presentations and disease course are regarded as the key indicators of the severity of inflammation for uveitis occurrence and treatment [12]. Therefore, we investigated the impact of ART on the onset, clinical presentations, and disease course of uveitis in rats. In our current clinical examination, ART pretreatment on rats can reduce the clinical scores on the recurrent point on days 18–19 post-immunization, although all rats developed anterior uveitis per slit biomedical microscopy, suggesting that ART might have a protective role in the treatment of relapsing uveitis. Other studies have also supported our conclusion in endotoxin-induced uveitis in rats [9] and in experimental autoimmune encephalomyelitis [5], rheumatoid arthritis [13,14], and sepsis. Furthermore, the present histopathological results in our study confirmed that ART pretreatment can suppress inflammation in the anterior chamber, interior segment, and retinal lesions of rats with relapsing uveitis. Our clinical and pathological results support the opinion that ART has suppressive effects on the pathogenesis and progression of chronic and relapsing uveitis.

The results of our metabolic profiles found 16 differently enriched metabolites, including pantothenate and CoA biosynthesis; aminoacyl-tRNA biosynthesis; alanine, aspartate, and glutamate metabolism; and arginine and proline metabolism between tEAU rats and healthy control rats, suggesting that the presence of distinct metabolic patterns in rats with relapsing uveitis compared with healthy control rats. Particularly, the most significant pathway in our study was pantothenate and CoA biosynthesis, which involves increased pantothenic acid, ureidopropionic acid, and D-4′-phosphopantothenate levels for tEAU onset. A recent similar report by Yang et al. revealed enriched pantothenate and CoA biosynthesis in patients with Vogt–Koyanagi–Harada (VKH) disease compared with control patients [15]. In addition, similar results of enriched pantothenate and CoA biosynthesis were also recorded in a mouse model of atopic dermatitis [16]. Several studies have revealed that the pantothenate and CoA biosynthesis pathway is related to amino acid metabolism, whereby branched-chain amino acids provide CoA derivatives to enter the tricarboxylic acid cycle [17,18]. Therefore, we speculated that various autoimmune disorders, including uveitis, in human and animal models might share metabolic regulation pathways and could be driven by common immunopathological mechanisms.

In line with our results, Yang et al. reported that the aminoacyl-tRNA biosynthesis pathway is significantly changed in patients with both VKH and Behçet’s disease (BD) by comparison with controls [15]. Aminoacyl-tRNAs are the crucial substrates that transfer specific amino acids to integrate them into the polypeptide chain synthesized during translation [19,20]. The aminoacyl-tRNA biosynthesis pathway is related to various diseases, including several autoimmune disorders [21,22,23]. Previous studies have revealed that alanine is involved in early T-cell activation, and its deprivation might damage T-cell effector functions [24]. The results of the altered L-glutamine, L-tryptophan, and L-alanine levels in the tEAU model suggest that those altered metabolites might be involved in the pathogenesis of uveitis by modulating T-cell activation and its effector functions.

ART has been demonstrated to play an antimalarial role; elicit anticancer, antidiabetic, antiviral, and anti-autoimmune responses; and have anti-inflammatory activities. Particularly, ART plays an immunomodulatory role in various immune cells and cytokines of the immune system, but it shows distinct regulatory effects in different immune conditions. The main metabolic finding in the present study is that ART increased the expression level of L-alanine, referring to alanine, aspartate, and glutamate metabolism and aminoacyl-tRNA biosynthesis, and enhanced the levels of spermidine and N-acetyl putrescine associated with arginine and proline metabolism. Alanine, aspartate, and glutamate metabolism is known to be closely linked to D-amino acid metabolism, the citrate cycle, and arginine and proline metabolism. Previous studies have revealed that alanine, aspartate, and glutamate metabolism is involved in the lens metabolism in bovines [25]. L-alanine is a component of the peptide of peptidoglycan, an effector of the leucine-responsive regulatory protein, and an inhibitor of glutamine synthetase [26]. These results suggest that ART plays modulated roles by changing the expression level of L-alanine in alanine, aspartate, and glutamate metabolism and aminoacyl-tRNA biosynthesis and the expression levels of spermidine and N-acetyl putrescine in arginine and proline metabolism associated with T-cell activation and effector function.

A shared significant perturbation of the arginine and proline metabolism pathway in the tEAU group and SOD-treated group vs. ART-treated group were also observed. Of special interest, downregulated spermidine level in the SOD-treated group and downregulated N-acetyl putrescine and spermidine levels in the tEAU group, but upregulated levels in the ART group, were observed. Spermidine displays various effects, including anti-inflammatory and antioxidant activities, boosting the mitochondrial metabolic role and respiration, and improving proteostasis and chaperone activity. For instance, it has been reported that spermidine protects retinal pigment epithelial cells from oxidative stress [27]. Moreover, it was revealed that spermidine, or L-arginine, promoted regulatory T-cell–cell differentiation within the gut and reduced pathology in a model of T-cell transfer-induced colitis [28]. Spermidine has also been shown to suppress the activation of inflammatory dendritic cells (DCs), reduce the production of pro-inflammatory cytokines such as TNF-α, IL-6, and IL-17, and inhibit inflammatory responses by activating the FOXO3 pathway and promoting autophagy [29]. From these results, we can reason that ART plays a therapeutic role by boosting spermidine levels in arginine and proline metabolism pathways associated with T-cell differentiation and immune response [30]. Meanwhile, as an intermediate in polyamine metabolism, N-acetylputrescine may indirectly influence the progression of ocular inflammation by modulating overall polyamine levels and the generation of toxic metabolites [31]. Our results showed that increased spermidine and N-acetyl putrescine levels are associated with milder relapsing uveitis, suggesting that they might be a potential predictive therapeutic biomarker in ART treatment.

Based on metabolomics findings, we may clinically assess disease activity and treatment response by analyzing plasma metabolites (e.g., spermidine, N-acetylputrescine) to facilitate personalized therapy. Dynamic monitoring of metabolic profile changes before and after treatment could optimize the dosage and duration of artesunate [32,33]. In certain cases, combining antioxidants (e.g., N-acetylcysteine) may further enhance anti-inflammatory and antioxidant effects, mitigate oxidative stress damage, improve therapeutic outcomes, and reduce adverse effects.

## 5. Conclusions

Our study demonstrates that ART treatment can alleviate recurrent uveitis by altering the plasma metabolic characteristics associated with T-cell activation and differentiation, which might provide novel insights for potential therapeutic treatments.

## Data Availability

The original contributions presented in this study are included in the article/Supplementary Materials. Further inquiries can be directed to the corresponding author(s).

## Supplementary Materials

The following supporting information can be downloaded at https://www.mdpi.com/article/10.3390/biomedicines13040821/s1, **Figure S1**: Metabolomic profiling by hydrophilic interaction liquid chromatography–mass spectroscopy (HILIC-MS) and reversed-phase liquid chromatography–mass spectroscopy (RPLC-MS).; Table S1: Shared pathways among the tEAU, ART, and SOD-treated groups compared with their matched group.

## Abbreviations

The following abbreviations are used in this manuscript:

ART	Artesunate
EAU	Experimental autoimmune uveitis
IRBP	Inter-photoreceptor retinoid-binding protein
tEAU	Recurrent uveitis induced by IRBP R16 peptide-specific T-cells
SPF	Specific-pathogen-free
aEAU	Induction of antigen-induced uveitis
CFA	Complete Freund’s Adjuvant
APCs	Antigen-presenting cells
SOD	Sodium bicarbonate
QC	Quality control
UHPLC	Ultra-high performance liquid chromatography
TOF	Time-of-fight
MS	Mass spectrometry
PCA	Non-supervised principal component analysis
OPLS-DA	Orthogonal partial least-squares discriminant analysis
ANOVA	Analysis of variance
VKH	Vogt–Koyanagi–Harada
BD	Behçet’s disease
CTL	Healthy control group
