# Supplementary figures and images for: Plasma Metabolic Profiles of Chronic and Recurrent Uveitis Treated by Artesunate in Lewis Rats
Source: Biomedicines. 2025 Mar 28;13(4):821. doi: 10.3390/biomedicines13040821 (PMC12025074; doi:10.3390/biomedicines13040821)

## Slide 1
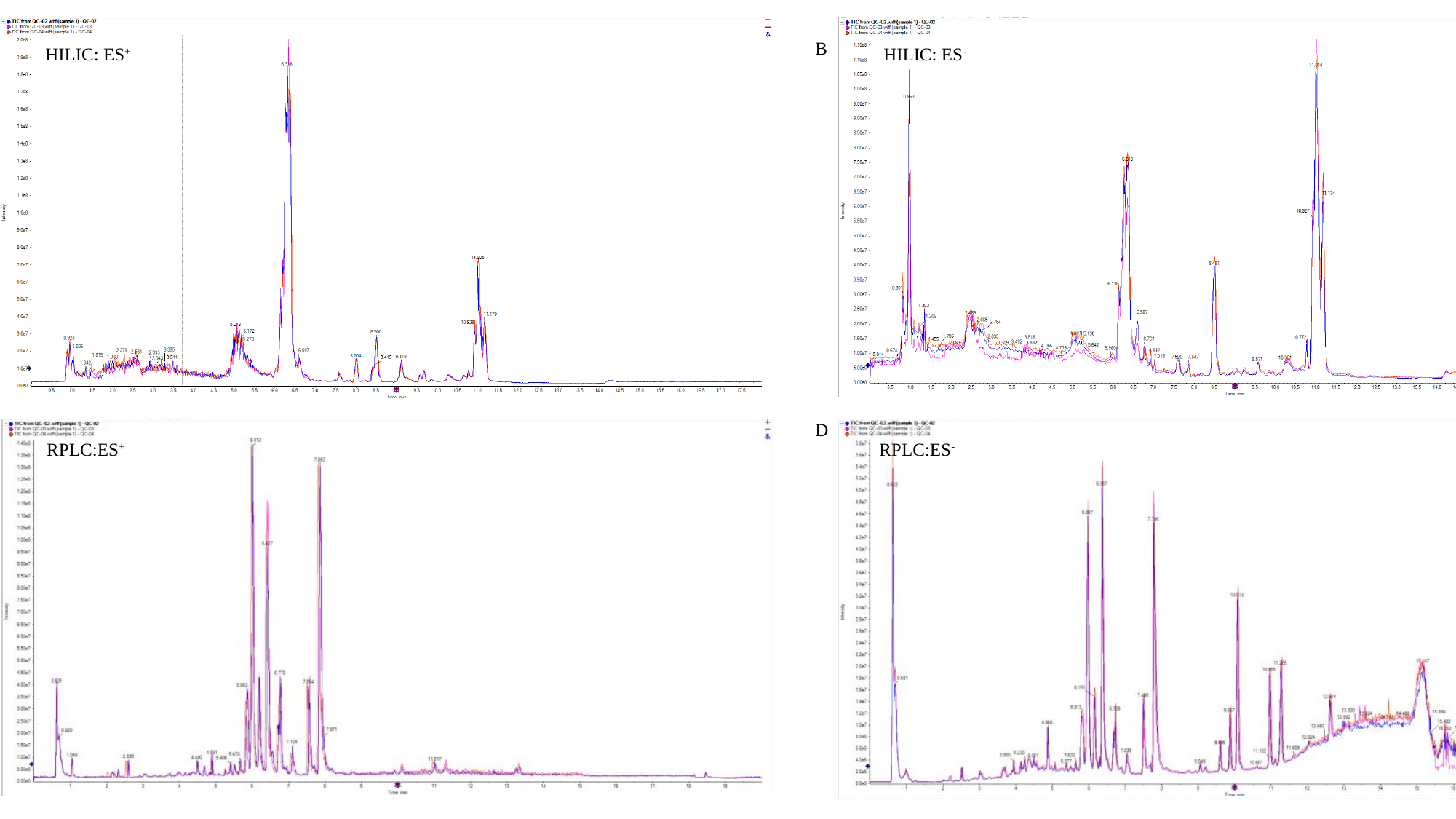

A
B
HILIC: ES+
HILIC: ES-
C
D
RPLC:ES+
RPLC:ES-

Supplement: Supplementary file 1 [file biomedicines-13-00821-s001.zip › Supplementary figure S1.pptx]
